# Long-term chronic conditions in individuals with mental and behavioural disorders: A data linkage study

**DOI:** 10.1177/00048674251315647

**Published:** 2025-02-05

**Authors:** Crystal Man Ying Lee, Kyran Graham-Schmidt, Kevin EK Chai, Daniel Rock, Suzanne Robinson, Mathew Coleman, Kim S Betts, Peter M McEvoy

**Affiliations:** 1 School of Population Health, Curtin University, Perth, WA, Australia; 2 Department of Health, Perth, WA, Australia; 3 WA Primary Health Alliance, Perth, WA, Australia; 4 Discipline of Psychiatry, Medical School, University of Western Australia, Perth, WA, Australia; 5 Faculty of Health, University of Canberra, Canberra, ACT, Australia; 6 Deakin Health Economics, Deakin University, Melbourne, VIC, Australia; 7 WA Country Health Service, Albany, WA, Australia; 8 Centre for Clinical Interventions, North Metropolitan Health Service, Perth, WA, Australia

**Keywords:** Mental health, physical health, comorbidity, health services

## Abstract

**Objective:**

This study aimed to investigate the physical health conditions among health service users in the first year since recorded mental disorder diagnosis in Western Australia.

**Methods:**

Community mental health, emergency department (ED) and inpatient records of individuals aged ⩾ 18 years with a recorded mental disorder diagnosis in state-funded health services were analysed. We identified long-term physical health conditions recorded within the first year of the first recorded mental disorder diagnosis. Prevalence of physical comorbidity across time was estimated using multinomial logistic regression. Mean number of health service contacts in the first year of the recorded mental disorder diagnosis was obtained using generalised linear model.

**Results:**

Altogether, 253,362 individuals were included. Within the first year of the first recorded mental disorder, the prevalence of at least one physical comorbidity ranged from 20.0% in 2006 to 14.5% in 2020. Cardiovascular disease was the most common comorbidity, but the most common combinations of comorbidities became more varied over time. The number of ED and inpatient contacts were higher in subgroups with a higher number of physical comorbidities (ED contacts: 2.4 [95% confidence intervals: 2.4, 2.4] for no comorbidities to 3.6 [3.4, 3.8] for ⩾ 3 comorbidities; inpatient contacts: 2.6 [2.6, 2.7] for no comorbidities to 4.5 [4.1, 4.9] for ⩾ 3 comorbidities).

**Conclusion:**

With a substantial proportion of individuals with mental disorders already having physical comorbidities on their first year of contact with state-funded health services, and the comorbidity combinations becoming more diverse, there is a need to implement more comprehensive joint mental and physical health services.

## Introduction

Mental and behavioural disorders are 1 of 10 long-term chronic conditions monitored periodically in Australia through the National Health Survey (Australian Bureau of Statistics [ABS], 2023a). There is accumulating evidence on the relationships of mental and behavioural disorders with physical health conditions, such as cardiovascular disease (Goldfarb et al., 2022) and physical multimorbidity (defined as any combination of chronic disease with at least one other acute or chronic disease; Pizzol et al., 2023). For cancer, while individuals with mental and behavioural disorders are not at increased risk of developing the condition, the risk of dying from cancer is higher than those without mental and behavioural disorders (Kim et al., 2022; Kisely et al., 2013). Nevertheless, for many individuals with mental and behavioural disorders, co-occurring physical health conditions are often considered as separate or siloed issues by health services and are managed accordingly, which may contribute to the poorer physical health of individuals with mental and behavioural disorders. Some other contributing factors to poorer physical health identified by consumers and experts in the Being Equally Well project report included fragmented care, absence of coordinated care, maldistribution of psychiatrists in rural and remote areas, and lack of information sharing between health services and health professionals (Morgan et al., 2021). Narrowed area of expertise through subspecialisation of the health workforce since the 1930s (Pashen et al., 2007), federated healthcare funding models (Australian Institute of Health and Welfare [AIHW], 2024a), and constraint in sharing of patient information (Parker et al., 2023) have also contributed to the separate management of mental and physical health conditions as opposed to a holistic approach. Indeed, higher rate of hospitalisation has been reported in people with both mental and physical health conditions than those with either condition or no condition (AIHW, 2012). Identification of such individuals for early intervention may, therefore, prevent potentially avoidable hospitalisations.

Globally, an estimate of 970 million individuals had mental and behavioural disorders in 2019 (GBD 2019 Mental Disorders Collaborators, 2022). A systematic review of 18 million individuals from 32 European countries estimated a 1% reduction in excess annual prevalence risks of physical health conditions equated to 2 million fewer physical health diagnoses associated with four common mental and behavioural disorders in 2019 (Wienand et al., 2024). Based on the estimates in the review, the authors argued for the value of improving integrated mental and physical healthcare approaches. Furthermore, a meta-analysis of 13 studies reported a higher prevalence of some physical health conditions among individuals with both severe mental illness and substance use disorders compared with those with severe mental illness alone (Onyeka et al., 2019). This underscored a need to consider physical health conditions and comorbid mental disorders.

The AIHW recently highlighted a need to study the impact of mental and physical health on health service usage across care settings (AIHW, 2024b). A Delphi study of people with lived or professional experience in mental health also identified physical health as a mental health research priority in Australia (McEvoy et al., 2024). In fact, the request for more research on mental and physical health conditions and integrated care in Australia began more than a decade ago (Happell et al., 2015). The review also called for research on the needs of people with mental and behavioural disorders in regional and remote areas as most studies have been conducted in major population centres along the eastern seaboard of Australia. With access to the linked health administrative records of individuals with a state-funded mental health service contact in Western Australia, we aimed to (1) determine the physical health conditions present among individuals on the first year of the first recorded mental and behavioural disorders diagnosis over time; (2) report on health service use by the number of physical comorbidities; (3) explore differences by geographical remoteness; and (4) explore differences by mental and behavioural disorder classes.

## Methods

We used data from a population-based mental health linkage project of health administrative records in Western Australia. Details of the linkage project have been published elsewhere (Lee et al., 2024). In brief, records of all individuals aged ⩾ 18 years with at least one mental health–related record since 1 January 2005 in the Hospital Morbidity Data Collection, Emergency Department (ED) Data Collection and the Mental Health Information Data Collection were linked with the Death Register. Linkage was carried out by the Data Linkage Services WA using probabilistic record linkage methods (Hodges et al., 2020). For this study, individuals were included for analysis if they had at least one recorded mental health service contact and a diagnosis of mental and behavioural disorders (International Statistical Classification of Diseases and Related Health Problems, 10th Revision, Australian Modification [ICD-10-AM]: F00-F99) based on all available diagnosis codes.

We restricted the physical health conditions to the nine long-term chronic conditions in the National Health Survey and defined these conditions based on the ICD-10-AM codes obtained from the AIHW, where available. These physical health conditions included arthritis (ICD-10-AM: M05–M06, M15–M19) (AIHW, 2023a), asthma (J45–J46) (AIHW, 2023b), back problems (M40–M54, M99) (AIHW, 2023a), cancer (malignant neoplasms; C00–C96), cardiovascular disease (heart, stroke and vascular disease; I00–I99) (AIHW, 2023c), chronic obstructive pulmonary disease (COPD; excluding asthma; J40–J44) (AIHW, 2023b), diabetes (including type 1, type 2 and type unknown; E10, E11, E14; AIHW, 2023d), kidney disease (N18, U87.1, Z49.1, Z49.2, Z94.0, Z99.2, T82.4, T86.1) and osteoporosis (M80–M82) (AIHW, 2023a). Physical health conditions were identified from all available diagnosis codes and grouped into four subgroups (none, one, two, and three or more comorbidities). Residential area of remoteness was classified as major city, inner regional, outer regional, remote and very remote according to the ABS classification (ABS, 2023b). Re-presentation of mental disorder was used as a proxy measure of mental disorder chronicity, which was defined as re-presentation with the same mental disorder diagnosis block (e.g. first recorded mental disorder diagnosis was F00–F09 and later re-presented with F00–F09) and included no re-presentation, re-presentation < 12 months and re-presentation ⩾ 12 months.

### Statistical analysis

We identified any physical health conditions recorded within the first year of the first recorded diagnosis of mental and behavioural disorders (i.e. all available records from the date of first recorded mental disorder diagnosis to 365 days after the first record). Since we were unable to ascertain whether mental and behavioural disorders were recorded prior to 2005, we excluded individuals whose first recorded diagnosis of mental and behavioural disorders occurred in 2005 in our study. Individuals whose first recorded diagnosis of mental and behavioural disorders occurred in 2021 were also excluded as the duration between their first recorded diagnosis and study end date was less than 1 year. Multinomial logistic regression was used to estimate the adjusted prevalence of physical comorbidity across time (2006–2020). The model was adjusted for age, sex, Aboriginal and Torres Strait Islander status, remoteness, year of first recorded mental and behavioural disorder diagnosis, and mental disorder re-presentation. The three most common comorbidity combinations for each comorbidity subgroup were reported from 2006 to 2020. For health service utilisation, we used generalised linear model with log link and gamma family to obtain adjusted mean number of health service contacts within each of the three care settings (i.e. community mental health, ED, and inpatient) in the first year since the recorded mental and behavioural disorders diagnosis for each comorbidity subgroup. Non-parametric bootstrapping was used to compute confidence intervals of the number of contacts. The model was adjusted for age, sex, Aboriginal and Torres Strait Islander status, remoteness, year of first recorded mental and behavioural disorder diagnosis, and mental disorder re-presentation. The analyses were also conducted by sex and by age (< 65 and ⩾ 65 years).

We repeated the analysis on the most common mental and behavioural disorder classes, which included substance use disorders (ICD-10-AM: F10–F19), psychotic disorders (F20–F29), affective disorders (F30–F39), anxiety disorders (F40–F41) and specific personality disorders (F60). Individuals were included in a mental disorder class if all records in the first week of their first recorded diagnosis of mental and behavioural disorders only included diagnosis codes in the same diagnosis block. For individuals with more than one mental disorder class on their first week of recorded diagnosis, they were included in the ‘comorbid mental disorders’ subgroup. Individuals without any of these mental disorder classes recorded on the first week of diagnosis were included in the subgroup ‘other mental disorders’. Analyses were performed using SAS 9.4 and Stata/MP V.18.0.

## Results

Altogether, 253,362 individuals with at least one recorded diagnosis of mental and behavioural disorders were included for analysis after excluding 32,392 individuals whose first recorded diagnosis occurred in 2005 and 16,694 individuals whose first recorded diagnosis occurred in 2021. At the first recorded diagnosis of mental and behavioural disorders, mean age (standard deviation) was 45.5 (22.5) years, 52.7% were females, and 64.0% lived in a major city (Table 1). Cardiovascular disease was the most prevalent physical health condition, whereby 7.6% of individuals recorded a relevant diagnosis in the 12 months after the first recorded mental and behavioural disorder diagnosis. Other physical health conditions ranged from 0.6% for asthma to 3.1% for back problems.

**Table 1.** Characteristics of individuals on their first recorded mental and behavioural disorder diagnosis.

	Current study
*N*	253,362
Mean (standard deviation) age (years)	45.5 (22.5)
Females	52.7%
Aboriginal and Torres Strait Islander status	
Yes	7.2%
No	90.3%
Missing	2.5%
Residential remoteness	
Major city	64.0%
Inner regional	15.5%
Outer regional	7.7%
Remote	4.4%
Very remote	4.6%
Missing	3.7%
Arthritis	1.3%
Asthma	0.6%
Back problems	3.1%
Cancer	1.5%
Cardiovascular disease	7.6%
Chronic obstructive pulmonary disease	1.6%
Diabetes	2.2%
Kidney disease	1.6%
Osteoporosis	0.8%

On the first year of the first recorded mental and behavioural disorders, most individuals did not have a recorded physical comorbidity. The adjusted prevalence of no physical comorbidities increased significantly over time from 80.0% (95% CI: [79.4%, 80.5%]) in 2006 to 85.5% [85.0%, 86.0%] in 2020 (Table 2). In contrast, the prevalence decreased significantly over the same period for one comorbidity (14.0% [13.5%, 14.5%] in 2006; 11.9% [11.4%, 12.4%] in 2020), two comorbidities (4.5% [4.2%, 4.8%] in 2006; 2.2% [2.0%, 2.4%] in 2020), and three or more comorbidities (1.6% [1.5%, 1.8%] in 2006; 0.4% [0.3%, 0.4%] in 2020). The proportion of individuals who died within the first year increased with increasing number of comorbidities (no comorbidities: 3.9%; one comorbidity: 11.8%; two comorbidities: 18.1%; three or more comorbidities: 20.7%). Furthermore, the group of individuals with no comorbidities was younger (mean age: 42–43 years) than those with at least one physical comorbidity and the number of comorbidities increased with age (one comorbidity: 59–61 years; two comorbidities: 68–71 years; three or more comorbidities: 72–76 years). When stratified by age, the prevalence of at least one comorbidity was significantly higher in the older (⩾ 65 years) compared to the younger (< 65 years) group (Table S1). There were no observable differences between the sex groups (Table S2).

**Table 2.** Prevalence of physical health comorbidities in the year of first recorded mental and behavioural disorders.

Year	*N*	No comorbidities		One comorbidity		Two comorbidities		Three or more comorbidities	
		M (SD) age, years	Prevalence^$^ (95% CI) (%)	M (SD) age, years	Prevalence^$^ (95% CI) (%)	M (SD) age, years	Prevalence^$^ (95% CI) (%)	M (SD) age, years	Prevalence^$^ (95% CI) (%)
2006	18,103	41.6 (19.3)	80.0 [79.4, 80.5]	59.1 (21.3)	14.0 [13.5, 14.5]	70.0 (16.7)	4.5 [4.2, 4.8]	74.7 (13.9)	1.6 [1.5, 1.8]
2007	16,015	41.8 (19.7)	81.6 [81.1, 82.2]	60.4 (21.3)	13.4 [12.9, 13.9]	69.6 (16.1)	3.9 [3.6, 4.2]	75.3 (13.4)	1.2 [1.0, 1.4]
2008	15,096	42.2 (20.2)	83.2 [82.6, 83.7]	60.7 (21.5)	12.5 [12.0, 13.0]	68.4 (17.7)	3.3 [3.0, 3.6]	72.8 (15.1)	1.0 [0.8, 1.1]
2009	14,767	42.5 (20.6)	84.3 [83.7, 84.8]	60.2 (21.8)	12.4 [11.8, 12.9]	68.7 (17.9)	2.7 [2.4, 3.0]	74.9 (13.1)	0.7 [0.5, 0.8]
2010	14,939	42.4 (21.0)	85.3 [84.8, 85.8]	61.4 (22.0)	11.9 [11.4, 12.4]	70.1 (16.9)	2.3 [2.1, 2.5]	75.0 (13.7)	0.5 [0.4, 0.6]
2011	15,508	42.6 (21.4)	84.8 [84.3, 85.3]	59.7 (22.2)	12.4 [11.9, 12.9]	70.5 (16.6)	2.4 [2.1, 2.6]	73.5 (14.7)	0.5 [0.4, 0.6]
2012	16,074	42.2 (21.3)	84.0 [83.5, 84.5]	60.3 (21.9)	13.3 [12.8, 13.8]	69.9 (16.5)	2.3 [2.0, 2.5]	74.8 (14.8)	0.5 [0.4, 0.6]
2013	16,295	42.4 (21.5)	84.3 [83.8, 84.8]	59.8 (22.3)	12.9 [12.4, 13.4]	69.7 (17.4)	2.3 [2.1, 2.5]	72.3 (15.0)	0.5 [0.4, 0.6]
2014	16,823	42.6 (21.6)	84.0 [83.5, 84.5]	60.4 (22.1)	13.0 [12.5, 13.5]	69.3 918.1)	2.5 [2.2, 2.7]	72.6 (12.9)	0.5 [0.4, 0.6]
2015	17,595	42.4 (21.7)	84.0 [83.5, 84.5]	60.6 (22.8)	12.9 [12.4, 13.4]	69.2 (18.1)	2.6 [2.4, 2.8]	75.1 (13.5)	0.5 [0.4, 0.6]
2016	18,442	42.7 (21.8)	84.0 [83.5, 84.5]	60.2 (23.0)	12.6 [12.1, 13.0]	70.4 (17.9)	2.8 [2.6, 3.0]	76.4 (11.7)	0.6 [0.5, 0.7]
2017	18,742	42.3 (21.7)	84.2 [83.7, 84.7]	60.6 (23.0)	12.9 [12.4, 13.4]	70.2 (18.3)	2.4 [2.2, 2.6]	73.6 (15.7)	0.5 [0.4, 0.6]
2018	18,545	42.1 (21.7)	84.6 [84.1, 85.1]	59.7 (23.6)	12.4 [11.9, 12.8]	68.9 (18.1)	2.5 [2.3, 2.7]	73.6 (16.1)	0.5 [0.4, 0.6]
2019	18,658	42.2 (21.8)	85.2 [84.7, 85.7]	59.0 (23.6)	12.5 [(12.0, 12.3]	69.4 (18.3)	1.9 [1.7, 2.1]	72.3 (15.0)	0.4 [0.3, 0.5]
2020	17,760	41.8 (21.7)	85.5 [85.0, 86.0]	59.2 (23.3)	11.9 [11.4, 12.4]	68.8 (19.2)	2.2 [2.0, 2.4]	72.3 (15.8)	0.4 [0.3, 0.4]

**Figure 1.**
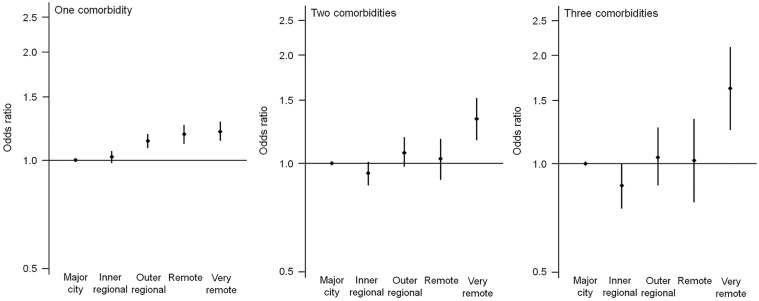
Odds ratios and 95% CIs of prevalent physical comorbidities associated with remoteness adjusted for age, sex, Aboriginal and Torres Strait Islander status, year of first recorded mental and behavioural disorder diagnosis, and mental disorder re-presentation.

$ Adjusted for age, sex, Aboriginal and Torres Strait Islander status, remoteness, year of first recorded mental and behavioural disorders diagnosis, and mental disorder re-presentation;

M = mean; SD = standard deviation; CI = confidence intervals;

Compared with individuals from a major city, a positive relationship was observed between one comorbidity and remoteness where the odds were 13–21% higher in outer regional, remote and very remote areas (Figure 1). For two and three or more comorbidities, the odds were only significantly higher in very remote compared with major city (two comorbidities: 1.37 [1.20, 1.57]; three or more comorbidities: 1.70 [1.30, 2.21]).

Of those with one comorbidity in the first year of the first recorded mental and behavioural disorders, cardiovascular disease was consistently the most common physical health condition (from 46.4% in 2006 to 38.2% in 2020) followed by back problems (from 16.3% in 2006 to 17.7% in 2020; Table 3). The third most common physical health condition was predominantly diabetes but was replaced by cancer, COPD and kidney disease in some years. For those with two comorbidities, the most common physical comorbidity combination was cardiovascular disease and diabetes, but the proportion of individuals with this combination at first recorded mental and behavioural disorders decreased over time (from 33.0% in 2006 to 12.2% in 2020; Table 3). Other combinations of significance were cardiovascular disease and back problems, cardiovascular disease and cancer, and cardiovascular disease and COPD. For those with three or more comorbidities, the most common physical comorbidity combination was cardiovascular disease, diabetes and kidney disease followed by cardiovascular disease, diabetes and cancer, and cardiovascular disease, diabetes and back problems (Table 3). Similar combinations were observed between the younger and older age groups and between females and males (Table S3).

**Table 3.** Most common comorbidity combinations at first recorded mental and behavioural disorders by number of physical health conditions.

Year	One comorbidity			Two comorbidities			Three or more comorbidities		
	Most common	Second	Third	Most common	Second	Third	Most common	Second	Third
2006	CVD (46.4%)	BP (16.3%)	DM (9.3%)	CVD/DM (33.0%)	CVD/BP (9.8%)	CVD/COPD (7.9%)	CVD/DM/KD (11.5%)	CVD/BP/DM (7.4%)	CVD/BP/osteoporosis (5.1%)
2007	CVD (45.0%)	BP (16.5%)	DM (8.0%)	CVD/DM (32.1%)	CVD/BP (9.9%)	CVD/COPD (9.3%)	CVD/DM/KD (12.4%)	CVD/BP/DM (9.2%)	CVD/cancer/DM (6.5%)
2008	CVD (46.4%)	BP (16.1%)	Cancer (8.1%)	CVD/DM (28.3%)	CVD/cancer (10.6%)	CVD/BP (9.6%)	CVD/DM/KD (10.3%)	CVD/BP/DM, CVD/cancer/DM and CVD/COPD/DM (6.2%)	
2009	CVD (46.0%)	BP (15.1%)	Cancer (7.5%)	CVD/DM (25.1%)	CVD/COPD (8.9%)	CVD/cancer (8.1%)	CVD/DM/KD (13.4%)	CVD/cancer/DM (10.3%)	CVD/BP/COPD (6.2%)
2010	CVD (43.2%)	BP (17.0%)	COPD (7.4%)	CVD/DM (15.3%)	CVD/cancer (12.6%)	CVD/BP (10.6%)	CVD/DM/KD (10.4%)	CVD/BP/KD (6.5%)	
2011	CVD (45.5%)	BP (16.6%)	Cancer (7.7%)	CVD/DM (15.8%)	CVD/BP (14.4%)	CVD/COPD (9.0%)	CVD/DM/KD (8.5%)	CVD/arthritis/BP and CVD/BP/cancer (7.0%)	
2012	CVD (40.8%)	BP (14.5%)	DM (13.5%)	CVD/DM (19.8%)	CVD/BP (9.9%)	CVD/cancer (7.7%)	CVD/DM/KD (12.8%)	CVD/BP/COPD and CVD/BP/DM (9.0%)	
2013	CVD (39.3%)	BP (16.2%)	DM (12.6%)	CVD/DM (17.7%)	CVD/BP (10.6%)	CVD/KD (9.0%)	CVD/DM/KD (12.6%)	CVD/cancer/COPD (6.9%)	CVD/COPD/DM (5.8%)
2014	CVD (40.1%)	BP (16.2%)	DM (10.7%)	CVD/BP (13.2%)	CVD/DM (12.7%)	CVD/KD (9.8%)	CVD/DM/KD (12.5%)	CVD/arthritis/DM (5.7%)	
2015	CVD (39.7%)	BP (17.0%)	DM (8.7%)	CVD/DM (11.4%)	CVD/COPD (10.1%)	CVD/BP (9.6%)	CVD/DM/KD & CVD/COPD/KD (6.6%)		CVD/cancer/DM (5.5%)
2016	CVD (40.0%)	BP (15.2%)	DM (9.2%)	CVD/BP (12.5%)	CVD/DM (11.6%)	CVD/COPD (9.9%)	CVD/BP/KD (5.5%)	CVD/cancer/DM, CVD/cancer/KD CVD/DM/KD & CVD/COPD/KD (4.6%)	
2017	CVD (38.0%)	BP (18.7%)	DM (9.0%)	CVD/BP (12.2%)	CVD/DM and CVD/KD (10.0%)		CVD/DM/KD and CVD/arthritis/BP (7.7%)		CVD/BP/osteoporosis CVD/cancer/KD and CVD/COPD/DM (5.5%)
2018	CVD (40.2%)	BP (17.7%)	KD (9.0%)	CVD/BP (13.4%)	CVD/DM (11.2%)	CVD/cancer (10.8%)	CVD/BP/osteoporosis (10.3%)	CVD/DM/KD (9.3%)	CVD/cancer/DM (6.2%)
2019	CVD (39.4%)	BP (17.3%)	KD (8.5%)	CVD/BP & CVD/DM (14.2%)		CVD/cancer (11.2%)	CVD/BP/cancer (9.2%)	CVD/COPD/DM (6.6%)	
2020	CVD (38.2%)	BP (17.7%)	DM (10.4%)	CVD/DM (12.2%)	CVD/KD (11.7%)	CVD/BP (10.8%)	CVD/DM/KD (13.3%)		

CVD: cardiovascular disease; BP: back problems; DM: diabetes; KD: kidney disease; COPD: chronic obstructive pulmonary disease.

**Figure 2.**
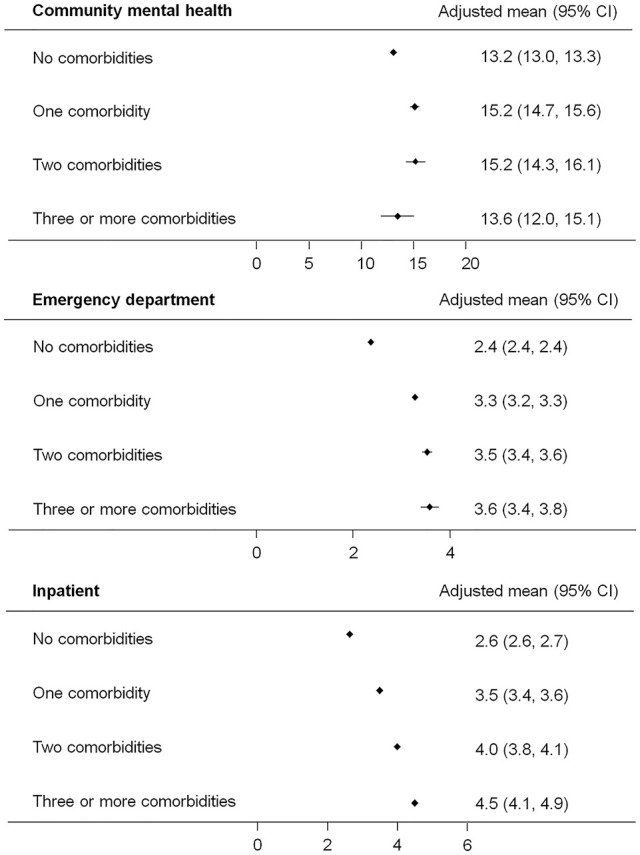
Mean number of health service contacts in the first year adjusted for age, sex, Aboriginal and Torres Strait Islander status, remoteness, year of first recorded mental and behavioural disorder diagnosis, and mental disorder re-presentation by care setting and number of physical comorbidities.

In the year of the first recorded mental and behavioural disorder diagnosis, the adjusted mean number of ED contacts was 2.4 (95% CI: [2.4, 2.4]) for individuals with no physical comorbidities (Figure 2). The number of contacts was higher for those with more comorbidities (e.g. 3.6 [3.4, 3.8] for three or more comorbidities). A similar pattern was observed for inpatient with the number of contacts ranging from 2.6 [2.6, 2.7] in those with no comorbidities to 4.5 [4.1, 4.9] in those with three or more comorbidities. The number of contacts in community mental health services was significantly higher in those with one (15.2 [14.7, 15.6]) and two (15.2 [14.3, 16.1]) compared to those with no comorbidities (13.2 [13.0, 13.3]). Of note, the number of ED contacts increased significantly with increasing remoteness (additional number of contacts relative to major city: ranged from 0.4 for inner regional to 1.4 for very remote; *p* < 0.001). In contrast, the number of community mental health service contacts decreased significantly with increasing remoteness (reduced number of contacts relative to major city: ranged from 0.7 for inner regional to 2.6 for very remote; *p* < 0.001). When stratified by age, lower number of community mental health service and ED contacts were observed in the older group with one or more comorbidities than the younger group with the same number of comorbidities (Table S4). In contrast, higher number of inpatient contacts were observed in the older group with no or one comorbidity than the younger equivalent. Females with one or two comorbidities had lower number of community mental health contacts than their male counterparts.

Similar patterns were observed between the most common mental and behavioural disorder classes where most individuals did not have physical comorbidities at first recorded mental and behavioural disorder diagnosis and the number of comorbidities increased with increasing age at first recorded diagnosis (Table S5). The difference in prevalence of comorbidities between the disorder classes was most likely influenced by the age at first recorded diagnosis. For example, 90.9% [89.9%, 91.9%] of individuals with specific personality disorders did not have physical comorbidities at first recorded diagnosis and the mean age in this group was 29.3 years. In comparison, 78.7% [78.5%, 79.0%] of individuals with other mental disorders did not have physical comorbidities at first recorded diagnosis and the mean age in this group was 51.7 years. The most common physical comorbidity combinations were similar between the mental disorder classes (Table S6). The number of community mental health service contacts was substantially higher in those with psychotic disorders, with and without physical comorbidities, compared with other mental disorder classes, most likely due to the nature of this particular class of mental and behavioural disorders (Table S7). The higher number of service contacts in individuals with specific personality disorders and three or more physical comorbidities should be interpreted with caution as the number of individuals in this subgroup was small as reflected in the wide confidence intervals.

## Discussion

In this large cohort of individuals with a recorded mental and behavioural disorders diagnosis in Western Australia, one-in-six individuals had at least one long-term physical health condition in their first year of diagnosis with cardiovascular disease being the most common condition. Not unexpectedly, the use of health services was higher in those with physical comorbidity and the number of contacts were generally higher in subgroups with a higher number of physical comorbidities. Importantly, the odds of having at least one physical comorbidity increased with increasing geographical remoteness from a major city.

Despite one-sixth of the cohort had physical comorbidities on the first year of recorded mental and behavioural disorder diagnosis, the proportion of individuals with at least one physical comorbidity on their first year of diagnosis appeared to have decreased in the 15-year period (2006–2020). While this seems encouraging, we need to acknowledge that, due to the nature of the dataset, our results are likely influenced by the number of service contacts (i.e. more service contacts mean more records available to identify comorbidities of interest). Therefore, it is possible that individuals with first mental health service contact in more recent time already had physical comorbidities in the first year, but the level of severity did not result in an ED presentation or hospital admission, which is a positive. Regardless, our results still suggest a substantial proportion of individuals with mental and behavioural disorders would benefit from early intervention to prevent future hospitalisation.

Of those with at least one physical comorbidity, cardiovascular disease was the most common physical condition in our cohort despite higher prevalences of physical conditions, such as arthritis and back problems, were observed in primary care setting. For example, an Australian general practice study of 173,861 patients aged ⩾ 15 years with severe or long-term mental illness reported 27% had arthritis, 22% had asthma, 35% had back pain, and 10% had cardiovascular disease (Belcher et al., 2021). This is largely due to physical conditions, such as cardiovascular disease and end-stage kidney disease, which require emergency or hospital treatment, being more likely to be captured in secondary and tertiary care health administrative data such as the one available for this project. Other physical conditions studied here are generally managed in primary care; hence, these conditions were not captured in our dataset unless they were related to the reason for the episode of care. Therefore, research that uses linked population-based primary, secondary and tertiary care data is essential to support population planning. Unfortunately, such linked data are not yet available for research in Australia. Nevertheless, the prevalence of cardiovascular disease in our cohort is noteworthy. Together with physical comorbidity combinations becoming more varied, there is a potential implication for prescribing psychotropic medications to people with chronic conditions, as they are most likely already taking multiple medications as recommended in clinical guidelines (National Heart Foundation of Australia, 2012; The Royal Australian College of General Practitioners, 2020). For example, the cardiovascular effect of psychotropic medications and the drug–drug interaction between psychotropic and cardiovascular medications has been documented (Pina et al., 2018). Similarly, interactions between psychotropics and anticancer medications have been reported (Yap et al., 2011). For individuals with renal impairment, dose adjustments of some psychotropics may be required (Cohen et al., 2004).

Our study reinforces the need to integrate mental and physical health services and improve the awareness of physical health within specialised mental health services. Unfortunately, it is not simply incomplete knowledge about what works that underpins the higher un- or undertreated morbidity and earlier mortality rates. It is also that we do not get close to the limits of the known avertable burden using what we do know but do not ensure happens. Many of the barriers to routine physical health treatments and better outcomes for individuals with severe mental illness are artefacts of the way local care systems are organised and funded rather than anything intrinsic to the nature of co-occurrence of the conditions themselves (The Royal Australian and New Zealand College of Psychiatrists, 2016) as initiatives like Equally Well in Australia (Roberts et al., 2018) and other programmes elsewhere (Stanley, 2020) have shown. The choice to organise care otherwise is an artificial barrier we can choose to dismantle.

Regarding health service use, a systematic review quantified the relationship between severe mental illness and health service utilisation for non-psychiatric medical disorders (Ronaldson et al., 2020). The odds of a non-psychiatric hospital admission were 84% higher (pooled odds ratio: 1.84 [1.21, 2.80]), and an ED presentation was 97% higher (1.97 [1.41, 2.76]), in patients with severe mental illness than patients without severe mental illness. Our study also found that the use of ED and inpatient services was higher in individuals with both mental and behavioural disorders and physical comorbidity than those with mental and behavioural disorders alone, and the number of service contacts increased with increasing number of comorbidities.

A finding that is possibly unique to populations similar to Western Australia, which consists of many remote locations, was the higher number of ED contacts and lower number of community mental health service contacts with increasing geographical remoteness away from the major city. Western Australia has a land area of 2.5 million km^2^ with 78.6% of the population residing in the only major city, which covers 0.1% of the state’s land area (ABS, 2023c). Limited mental health specialist services in regional and remote Western Australia (Kaleveld et al., 2023) due to their very low population density potentially force residents in these areas to access health care in EDs. An Australian study on aeromedical retrieval of people for mental health care identified 6 of the 10 highest population regions with limited mental health clinical services were in Western Australia (Gardiner et al., 2019). In addition to the differences in the number of service contacts by remoteness, the odds of having at least one physical comorbidity on the first year of the first recorded mental and behavioural disorders diagnosis increased with increasing geographical remoteness from major city. The odds of physical comorbidities were significantly higher in the very remote group. This is the result of a combination of higher prevalence of health risk factors, poorer health outcomes, and barriers to accessing health care in regional and remote Australia than in major cities (AIHW, 2023e).

The strength of our study was the population-wide coverage of adults with mental and behavioural disorders who accessed state-funded health services in Western Australia. Nevertheless, a few limitations warrant mention. First, we did not have records of individuals who did not have a recorded mental and behavioural disorder diagnosis for comparison. Characteristic differences between health administrative records and self-reported data also meant that we were unable to compare our results with those of the general population reported in the National Health Survey. Second, hospital procedure codes and cause of death data, which are relevant in the identification of physical health conditions, were not available. Third, the lack of primary care records meant that physical health conditions that are mainly managed in primary care settings were poorly captured in our data set. Fourth, only nine physical health conditions were studied; we acknowledge that there are other chronic conditions of importance, e.g. chronic liver disease specifically in the substance use disorders subgroup or neurological conditions, such as Parkinson’s disease. Fifth, considering contemporary approaches to data sovereignty and population descriptions, we did not seek Aboriginal health and research ethics approval to conduct sub-analysis for Aboriginal and Torres Strait Islander groups. Nevertheless, the investigation of priority populations is an important area for future research.

This is the first step in studying mental and physical health conditions on health service usage in Western Australia. With a substantial proportion of individuals with mental and behavioural disorders on their first year of contact with state-funded health services already having other long-term chronic conditions, and the combination of chronic conditions becoming more diverse, this information could influence how patients with both mental and physical health conditions should be managed. More broadly, there is a need to implement more comprehensive joint mental and physical health services or at least coordinated care where patient information can be shared between healthcare providers with the patient’s consent. In terms of research, having access to linked primary, secondary and tertiary care data will further support research and population planning that focuses on the whole person (i.e. both mental and physical health).

## Supplemental Material

sj-pdf-1-anp-10.1177_00048674251315647 – Supplemental material for Long-term chronic conditions in individuals with mental and behavioural disorders: A data linkage study Supplemental material, sj-pdf-1-anp-10.1177_00048674251315647 for Long-term chronic conditions in individuals with mental and behavioural disorders: A data linkage study by Crystal Man Ying Lee, Kyran Graham-Schmidt, Kevin EK Chai, Daniel Rock, Suzanne Robinson, Mathew Coleman, Kim S Betts and Peter M McEvoy in Australian & New Zealand Journal of Psychiatry
