# Supplementary material for: Long-term chronic conditions in individuals with mental and behavioural disorders: A data linkage study
Source: Aust N Z J Psychiatry. 2025 Feb 5;59(3):260–9. doi: 10.1177/00048674251315647 (PMC11837424; doi:10.1177/00048674251315647)
Supplement: sj-pdf-1-anp-10.1177_00048674251315647 – Supplemental material for Long-term chronic conditions in individuals with mental and behavioural disorders: A data linkage study [file sj-pdf-1-anp-10.1177_00048674251315647.pdf]

Long-term chronic conditions in individuals with mental and behavioural disorders: A data-linkage study

Crystal Man Ying Lee, Kyran Graham-Schmidt, Kevin E.K. Chai, Daniel Rock, Suzanne Robinson, Mathew Coleman, Kim S. Betts, Peter M. McEvoy

Table S1: Prevalence[$] (95% confidence intervals) of physical health comorbidities in the year of first recorded mental and behavioural disorders by age

Year	No comorbidities		One comorbidity		Two comorbidities		Three or more comorbidities	
	Age <65 year	Age ≥65 years	Age <65 year	Age ≥65 years	Age <65 year	Age ≥65 years	Age <65 year	Age ≥65 years
2006	88.7 (88.2, 89.1)	52.7 (51.2, 54.3)	9.4 (8.9, 9.8)	28.1 (26.7, 29.5)	1.6 (1.4, 1.8)	13.6 (12.5, 14.6)	0.4 (0.3, 0.5)	5.6 (4.9, 6.3)
2007	89.8 (89.3, 90.3)	55.8 (54.2, 57.4)	8.5 (8.0, 9.0)	28.7 (27.2, 30.1)	1.5 (1.3, 1.7)	11.5 (10.5, 12.5)	0.3 (0.2, 0.4)	4.0 (3.4, 4.7)
2008	90.4 (89.8, 90.9)	60.6 (58.9, 62.2)	7.9 (7.4, 8.4)	27.1 (25.6, 28.5)	1.5 (1.3, 1.7)	9.1 (8.1, 10.0)	0.3 (0.2, 0.3)	3.3 (2.7, 3.9)
2009	90.8 (90.3, 91.3)	63.6 (62.0, 65.2)	8.0 (7.5, 8.4)	26.2 (24.7, 27.7)	1.1 (0.9, 1.2)	7.8 (6.9, 8.8)	0.1 (0.1, 0.2)	2.3 (1.9, 2.9)
2010	91.6 (91.1, 92.1)	65.2 (63.6, 66.7)	7.4 (6.9, 7.8)	26.2 (24.8, 27.7)	0.9 (0.7, 1.1)	6.7 (5.9, 7.6)	0.1 (0.1, 0.2)	1.9 (1.4, 2.3)
2011	90.3 (89.8, 90.8)	67.4 (65.9, 68.9)	8.5 (8.0, 9.0)	24.6 (23.3, 26.0)	1.0 (0.8, 1.2)	6.5 (5.7, 7.3)	0.1 (0.1, 0.2)	1.4 (1.1, 1.8)
2012	90.0 (89.5, 90.5)	64.9 (63.4, 66.4)	9.0 (8.5, 9.5)	26.9 (25.6, 28.3)	0.9 (0.7, 1.1)	6.6 (5.8, 7.4)	0.1 (0.1, 0.2)	1.6 (1.2, 2.0)
2013	89.8 (89.3, 90.3)	66.8 (65.3, 68.3)	9.0 (8.4, 9.5)	25.4 (24.0, 26.8)	1.1 (0.9, 1.3)	6.1 (5.4, 6.9)	0.2 (0.1, 0.2)	1.7 (1.3, 2.1)
2014	89.5 (89.0, 90.1)	66.2 (64.8, 67.7)	9.0 (8.6, 9.6)	25.7 (24.3, 27.0)	1.2 (1.0, 1.4)	6.6 (5.8, 7.3)	0.2 (0.1, 0.3)	1.5 (1.2, 1.9)
2015	89.8 (89.3, 90.3)	65.1 (63.7, 66.5)	8.9 (8.4, 9.3)	26.0 (24.7, 27.3)	1.2 (1.0, 1.4)	7.2 (6.4, 7.9)	0.1 (0.1, 0.2)	1.7 (1.3, 2.1)
2016	89.6 (89.1, 90.1)	65.7 (64.3, 67.0)	9.0 (8.5, 9.4)	24.5 (23.3, 25.8)	1.3 (1.1, 1.5)	7.9 (7.1, 8.6)	0.1 (0.1, 0.2)	2.0 (1.6, 2.4)
2017	90.1 (89.6, 90.6)	65.3 (63.9, 66.7)	8.7 (8.2, 9.2)	26.6 (25.3, 27.8)	1.1 (0.9, 1.2)	6.7 (6.0, 7.5)	0.2 (0.1, 0.3)	1.4 (1.0, 1.7)
2018	90.0 (89.5, 90.5)	66.9 (65.5, 68.3)	8.6 (8.2, 9.1)	24.7 (23.4, 25.9)	1.2 (1.0, 1.4)	6.7 (6.0, 7.4)	0.2 (0.1, 0.2)	1.7 (1.3, 2.1)
2019	90.1 (89.6, 90.6)	69.4 (68.0, 70.7)	8.9 (8.4, 9.4)	24.2 (23.0, 25.5)	0.9 (0.7, 1.1)	5.1 (4.4, 5.7)	0.1 (0.1, 0.2)	1.3 (1.0, 1.6)
2020	90.5 (90.0, 91.0)	69.2 (67.8, 70.7)	8.3 (7.8, 8.8)	23.7 (22.4, 25.1)	1.1 (0.9, 1.3)	5.9 (5.2, 6.6)	0.1 (0.1, 0.2)	1.1 (0.8, 1.4)

[$] Adjusted for age, sex, Aboriginal and/or Torres Strait Islander status, remoteness, year of first recorded mental and behavioural disorders diagnosis, and mental disorder re-presentation;

Table S2: Prevalence$^{\$}$ (95% confidence intervals) of physical health comorbidities in the year of first recorded mental and behavioural disorders by sex

Year	No comorbidities		One comorbidity		Two comorbidities		Three or more comorbidities	
	Female	Male	Female	Male	Female	Male	Female	Male
2006	80.5 (79.8, 81.2)	79.3 (78.5, 80.1)	13.6 (13.0, 14.3)	14.3 (13.6, 15.1)	4.2 (3.8, 4.6)	4.8 (4.4, 5.3)	1.7 (1.4, 1.9)	1.6 (1.3, 1.9)
2007	81.8 (81.0, 82.5)	81.4 (80.6, 82.2)	13.5 (12.8, 14.2)	13.2 (12.4, 13.9)	3.7 (3.4, 4.2)	4.1 (3.6, 4.5)	1.0 (0.8, 1.2)	1.4 (1.1, 1.6)
2008	83.6 (82.8, 84.3)	82.7 (81.9, 83.5)	12.4 (11.7, 13.1)	12.7 (11.9, 13.5)	3.2 (2.8, 3.5)	3.5 (3.1, 4.0)	0.9 (0.7, 1.1)	1.1 (0.8, 1.3)
2009	84.8 (84.1, 85.5)	83.7 (82.8, 84.5)	11.9 (11.2, 12.6)	12.9 (12.1, 13.7)	2.7 (2.3, 3.0)	2.8 (2.4, 3.1)	0.7 (0.5, 0.8)	0.7 (0.5, 0.9)
2010	85.7 (84.9, 86.4)	84.9 (84.0, 85.7)	11.7 (11.0, 12.3)	12.1 (11.4, 12.9)	2.2 (1.8, 2.5)	2.2 (2.1, 2.8)	0.5 (0.4, 0.7)	0.5 (0.4, 0.7)
2011	85.1 (84.3, 85.8)	84.4 (83.7, 85.2)	12.1 (11.5, 12.8)	12.7 (12.0, 13.5)	2.3 (2.0, 2.7)	2.4 (2.0, 2.7)	0.4 (0.3, 0.6)	0.5 (0.3, 0.6)
2012	84.3 (83.6, 85.0)	83.6 (82.9, 84.4)	12.9 (12.2, 13.6)	13.7 (12.9, 14.4)	2.4 (2.1, 2.7)	2.1 (1.8, 2.4)	0.4 (0.3, 0.6)	0.5 (0.4, 0.7)
2013	84.8 (84.1, 85.6)	83.7 (83.0, 84.5)	12.5 (11.8, 13.2)	13.3 (12.6, 14.0)	2.3 (2.0, 2.6)	2.3 (2.0, 2.6)	0.4 (0.3, 0.5)	0.7 (0.5, 0.8)
2014	84.3 (83.6, 85.0)	83.7 (83.0, 84.5)	12.9 (12.2, 13.6)	13.2 (12.4, 13.9)	2.4 (2.1, 2.7)	2.5 (2.2, 2.9)	0.4 (0.3, 0.6)	0.6 (0.4, 0.8)
2015	84.3 (83.6, 85.0)	83.7 (82.9, 84.4)	12.5 (11.9, 13.2)	13.3 (12.6, 14.0)	2.7 (2.3, 3.0)	2.5 (2.2, 2.9)	0.5 (0.4, 0.6)	0.5 (0.4, 0.7)
2016	83.8 (83.1, 84.5)	84.2 (83.5, 84.9)	12.6 (12.0, 13.3)	12.6 (11.9, 13.2)	2.9 (2.6, 3.3)	2.7 (2.4, 3.0)	0.6 (0.4, 0.7)	0.5 (0.4, 0.7)
2017	84.2 (83.5, 84.8)	84.3 (83.6,)85.0	12.9 (12.3, 13.6)	12.9 (12.2, 13.6)	2.4 (2.1, 2.7)	2.4 (2.1, 2.7)	0.5 (0.4, 0.7)	0.4 (0.3, 0.5)
2018	84.2 (83.5, 84.9)	85.0 (84.3, 85.7)	12.7 (12.1, 13.4)	12.0 (11.4, 12.7)	2.6 (2.2, 2.9)	2.4 (2.1, 2.7)	0.5 (0.4, 0.7)	0.5 (0.4, 0.7)
2019	84.9 (84.2, 85.6)	85.7 (85.0, 86.4)	13.0 (12.3, 13.6)	11.9 (11.2, 12.6)	1.8 (1.5, 2.0)	2.0 (1.7, 2.3)	0.4 (0.2, 0.5)	0.5 (0.3, 0.6)
2020	85.3 (84.6, 86.0)	85.8 (85.1, 86.5)	12.1 (11.5, 12.8)	11.7 (11.0, 12.3)	2.1 (1.8, 2.4)	2.3 (1.9, 2.6)	0.4 (0.3, 0.6)	0.3 (0.2, 0.4)

$^{\$}$ Adjusted for age, Aboriginal and/or Torres Strait Islander status, remoteness, year of first recorded mental and behavioural disorders diagnosis, and mental disorder re-presentation;

Table S3: Physical comorbidities at first recorded mental and behavioural disorders in 2006-2020 by age group and sex

Subgroup	Most common physical comorbidity combinations		
	One	Two	Three or more
Age <65 years	CVD (46.2%)	CVD/DM (26.2%)	CVD/DM/KD (10.6%)
	BP (21.4%)	CVD/BP (16.8%)	CVD/BP/DM (8.9%)
	DM (10.9%)	CVD/cancer (9.4%)	CVD/cancer/DM (8.4%)
Age ≥65 years	CVD (37.2%)	CVD/DM (15.8%)	CVD/DM/KD (10.1%)
	KD (12.0%)	CVD/KD (9.1%)	CVD/BP/osteoporosis (4.4%)
	BP (11.4%)	CVD/BP (8.1%)	CVD/cancer/DM (4.0%)
Female	CVD (41.5%)	CVD/DM (17.1%)	CVD/DM/KD (8.4%)
	BP (17.9%)	CVD/BP (12.3%)	CVD/BP/osteoporosis (6.3%)
	DM (8.0%)	CVD/cancer (7.9%)	CVD/BP/DM (5.1%)
Male	CVD (41.2%)	CVD/DM (21.6%)	CVD/DM/KD (12.1%)
	BP (15.0%)	CVD/COPD (9.6%)	CVD/cancer/DM (6.6%)
	DM (10.0%)	CVD/BP (9.6%)	CVD/BP/DM & CVD/COPD/DM (4.6%)

CVD = cardiovascular disease; BP = back problems; COPD = chronic obstructive pulmonary disease; DM = diabetes; KD = kidney disease;

Table S4: Mean number of health service contacts in the first year adjusted for age, sex, Aboriginal and/or Torres Strait Islander status, remoteness, year of first recorded mental and behavioural disorder diagnosis, and metal disorder re-presentation by care setting, number of physical comorbidities, age group and sex

Subgroup	Number of contacts (95% confidence intervals)			
	Number of physical comorbidities			
	None	One	Two	Three or more
**Community mental health**				
Age <65 years	13.1 (13.0, 13.3)	16.4 (15.9, 16.9)	17.1 (15.7, 18.5)	15.4 (11.6, 19.3)
Age ≥65 years	13.5 (13.1, 13.9)	12.8 (12.3, 13.3)	13.4 (12.4, 14.4)	12.4 (10.7, 14.1)
Female	13.8 (13.6, 14.0)	16.2 (15.6, 16.8)	16.5 (15.3, 17.8)	13.8 (11.9, 15.7)
Male	12.4 (12.2, 12.6)	14.0 (13.5, 14.6)	13.8 (12.7, 14.9)	13.3 (11.0, 15.6)
**Emergency department**				
Age <65 years	2.4 (2.4, 2.4)	3.7 (3.6, 3.8)	4.4 (4.1, 4.6)	4.6 (4.0, 5.3)
Age ≥65 years	2.3 (2.3, 2.4)	2.7 (2.7, 2.8)	3.0 (2.9, 3.1)	3.1 (3.0, 3.3)
Female	2.4 (2.3, 2.4)	3.3 (3.2, 3.3)	3.6 (3.4, 3.8)	3.6 (3.4, 3.8)
Male	2.4 (2.3, 2.4)	3.3 (3.2, 3.3)	3.4 (3.3, 3.6)	3.5 (3.3, 3.8)
**Inpatient**				
Age <65 years	2.2 (2.2, 2.2)	3.3 (3.2, 3.4)	4.4 (4.1, 4.7)	6.0 (4.8, 7.3)
Age ≥65 years	3.7 (3.6, 3.8)	4.1 (4.0, 4.2)	4.6 (4.4, 4.7)	5.1 (4.8, 5.4)
Female	2.6 (2.5, 2.6)	3.4 (3.3, 3.5)	4.0 (3.8, 4.1)	4.2 (3.8, 4.6)
Male	2.7 (2.7, 2.8)	3.5 (3.4, 3.7)	4.0 (3.8, 4.3)	4.9 (4.1, 5.6)

Table S5: Prevalence of physical health comorbidities in the year of first recorded mental and behavioural disorders in 2006-2020 by mental disorder class

Mental disorder class	N (%)	No comorbidities		One comorbidity		Two comorbidities		≥ Three comorbidities	
		Mean (SD) age years	Prevalence$^{\$}$ (95% CI) (%)	Mean (SD) age years	Prevalence$^{\$}$ (95% CI) (%)	Mean (SD) age years	Prevalence$^{\$}$ (95% CI) (%)	Mean (SD) age years	Prevalence$^{\$}$ (95% CI) (%)
Substance use disorders	61,634 (24.3)	35.1 (15.9)	85.6 (85.3, 88.0)	50.4 (18.9)	11.4 (11.1, 11.6)	60.5 (16.2)	2.5 (2.3, 2.6)	66.1 (14.2)	0.6 (0.5, 0.6)
Psychotic disorders	8024 (3.2)	40.3 (18.1)	86.6 (85.9, 87.3)	53.3 (21.1)	10.5 (9.9, 11.2)	63.3 (17.9)	2.3 (2.0, 2.7)	71.0 (11.5)	0.6 (0.4, 0.8)
Affective disorders	41,623 (16.4)	42.3 (19.4)	85.3 (85.0, 85.6)	57.4 (21.0)	11.7 (11.4, 12.0)	67.6 (17.3)	2.4 (2.2, 2.5)	73.4 (14.0)	0.6 (0.6, 0.7)
Anxiety disorders	39,738 (15.7)	41.4 (19.2)	87.6 (87.3, 87.9)	55.4 (21.4)	10.3 (10.0, 10.6)	65.5 (17.8)	1.7 (1.6, 1.8)	71.2 (15.6)	0.4 (0.3, 0.4)
Specific personality disorders	3191 (1.3)	29.3 (12.9)	90.9 (89.9, 91.9)	36.7 (20.3)	8.1 (7.2, 9.0)	51.1 (24.4)	1.0 (0.7, 1.4)	-	-
Comorbid mental disorders (i.e. combination of the above disorders)	21,976 (8.7)	36.0 (15.2)	86.0 (85.6, 86.4)	46.3 (18.2)	11.8 (11.4, 12.2)	59.0 (17.1)	1.8 (1.6, 2.0)	64.8 (15.7)	0.4 (0.3, 0.4)
Other (i.e. none of the above disorders)	77,176 (30.5)	51.7 (25.5)	78.7 (78.5, 79.0)	71.7 (20.3)	16.3 (16.1, 16.6)	77.5 (14.2)	4.0 (3.8, 4.1)	79.9 (10.9)	1.0 (0.9, 1.1)

$^{\$}$ Adjusted for age, sex, Aboriginal and/or Torres Strait Islander status, remoteness, year of first recorded mental and behavioural disorders diagnosis, and mental disorder re-presentation;

SD = standard deviation; CI = confidence intervals

Table S6: Physical comorbidities at first recorded mental and behavioural disorders in 2006-2020 by mental disorder class

Mental disorder class (ICD-10-AM)	Most common physical comorbidity combinations		
	One	Two	Three or more
Substance use disorders (F10-F19)	CVD (44.6%)<br>BP (13.6%)<br>COPD (12.6%)	CVD/DM (21.2%)<br>CVD/COPD (15.1%)<br>CVD/BP (9.8%)	CVD/BP/COPD (45.4%)<br>CVD/DM/KD (6.7%)<br>CVD/COPD/KD & CVD/DM/KD (5.9%)
Psychotic disorders (F20-F29)	CVD (50.7%)<br>BP (14.8%)<br>DM (13.1%)	CVD/DM (32.6%)<br>CVD/BP (13.4%)<br>CVD/cancer (9.1%)	CVD/DM/KD (18.6%)
Affective disorders (F30-F39)	CVD (38.3%)<br>BP (21.0%)<br>DM (10.6%)	CVD/DM (18.4%)<br>CVD/BP (13.2%)<br>CVD/cancer (7.8%)	CVD/DM/KD (10.3%)<br>CVD/BP/DM (8.1%)<br>CVD/BP/arthritis (5.8%)
Anxiety disorders (F40-F41)	CVD (41.3%)<br>BP (21.2%)<br>Cancer (7.9%)	CVD/DM (15.7%)<br>CVD/BP (14.2%)<br>CVD/cancer (10.8%)	CVD/DM/KD (8.3%)<br>CVD/BP/DM & CVD/cancer/DM (5.5%)
Specific personality disorders (F60)	CVD (43.8%)<br>BP (28.7%)<br>DM (8.1%)	CVD/BP (27.6%)<br>CVD/DM (17.2%)	
Comorbid mental disorders (i.e. combination of the above disorders)	CVD (46.7%)<br>BP (23.0%)<br>DM (9.7%)	CVD/DM (22.1%)<br>CVD/BP (20.3%)<br>CVD/COPD (8.5%)	CVD/DM/arthritis (7.5%)<br>CVD/cancer/DM & CVD/DM/KD (6.3%)
Other (i.e. none of the above disorders)	CVD (40.1%)<br>BP (13.5%)<br>CKD (11.0%)	CVD/DM (18.2%)<br>CVD/KD (10.3%)<br>CVD/BP & CVD/cancer (8.7%)	CVD/DM/KD (12.6%)<br>CVD/BP/arthritis & CVD/BP/DM (4.7%)

ICD-10-AM: International Statistical Classification of Diseases and Related Health Problems, Tenth Revision, Australian Modification; CVD = cardiovascular disease; BP = back problems; COPD = chronic obstructive pulmonary disease; DM = diabetes; KD = kidney disease;

Table S7: Mean number of health service contacts in the first year adjusted for age, sex, Aboriginal and/or Torres Strait Islander status, remoteness, year of first recorded mental and behavioural disorder diagnosis, and metal disorder re-presentation by care setting, number of physical comorbidities, and mental disorder class

Mental disorder class (ICD-10-AM)	Number of contacts (95% confidence intervals)			
	Number of physical comorbidities			
	None	One	Two	Three or more
**Community mental health**				
Substance use disorders (F10-F19)	7.3 (7.1, 7.5)	8.1 (7.5, 8.6)	8.4 (7.3, 9.5)	8.4 (6.6, 10.2)
Psychotic disorders (F20-F29)	26.9 (26.0, 27.7)	31.5 (29.0, 34.0)	36.3 (29.6, 43.1)	16.9 (11.9, 21.8)
Affective disorders (F30-F39)	14.2 (13.9, 14.5)	17.0 (16.2, 17.9)	18.3 (16.3, 20.2)	17.4 (13.6, 21.2)
Anxiety disorders (F40-F41)	11.3 (11.0, 11.6)	14.2 (13.2, 15.2)	13.8 (11.3, 16.3)	13.7 (4.8, 22.7)
Specific personality disorders (F60)	18.4 (17.5, 19.4)	27.4 (23.0, 31.8)	24.6 (15.3, 33.8)	57.8 (16.2, 99.4)
Comorbid mental disorders	17.6 (17.2, 18.1)	20.0 (18.8, 21.3)	20.2 (17.4, 22.9)	24.1 (16.5, 31.7)
Other disorders	10.3 (10.1, 10.5)	11.9 (11.4, 12.5)	11.6 (10.4, 12.7)	9.7 (8.3, 11.0)
**Emergency department**				
Substance use disorders (F10-F19)	2.5 (2.5, 2.5)	3.5 (3.4, 3.6)	3.8 (3.6, 4.0)	4.3 (3.8, 4.8)
Psychotic disorders (F20-F29)	2.1 (2.1, 2.2)	2.7 (2.5, 2.8)	3.1 (2.5, 3.7)	2.1 (1.6, 2.6)
Affective disorders (F30-F39)	2.2 (2.2, 2.3)	3.0 (2.9, 3.1)	3.6 (3.1, 4.0)	3.2 (2.9, 3.5)
Anxiety disorders (F40-F41)	2.3 (2.2, 2.3)	3.5 (3.4, 3.7)	4.1 (3.8, 4.5)	3.2 (2.7, 3.7)
Specific personality disorders (F60)	3.0 (2.8, 3.2)	5.8 (4.6, 6.9)	7.7 (2.1, 13.2)	27.1 (11.3, 42.9)
Comorbid mental disorders	2.6 (2.5, 2.6)	3.7 (3.5, 3.8)	4.6 (3.9, 5.3)	4.7 (3.9, 5.6)
Other disorders	2.3 (2.3, 2.3)	3.0 (2.9, 3.0)	3.0 (2.9, 3.1)	3.3 (3.1, 3.5)
**Inpatient**				
Substance use disorders (F10-F19)	2.2 (2.1, 2.2)	3.2 (3.1, 3.4)	4.0 (3.7, 4.3)	5.3 (4.2, 6.5)
Psychotic disorders (F20-F29)	2.2 (2.0, 2.4)	3.0 (2.6, 3.4)	2.9 (2.6, 3.3)	3.2 (2.6, 3.8)
Affective disorders (F30-F39)	2.7 (2.6, 2.8)	3.6 (3.4, 3.8)	4.2 (3.7, 4.7)	4.2 (3.6, 4.8)
Anxiety disorders (F40-F41)	2.6 (2.5, 2.7)	3.5 (3.3, 3.7)	4.7 (4.0, 5.3)	5.3 (3.0, 7.5)
Specific personality disorders (F60)	2.4 (2.1, 2.6)	4.0 (3.3, 4.7)	4.8 (3.0, 6.6)	7.2 (<0.1, 15.2)
Comorbid mental disorders	2.3 (2.2, 2.3)	3.3 (3.2, 3.5)	4.6 (3.9, 5.2)	4.6 (3.9, 5.2)
Other disorders	3.2 (3.1, 3.3)	3.8 (3.7, 4.0)	4.1 (3.9, 4.3)	4.6 (4.2, 5.0)

ICD-10-AM: International Statistical Classification of Diseases and Related Health Problems, Tenth Revision, Australian Modification
